# Supplementary material for: Yield-Related QTL Clusters and the Potential Candidate Genes in Two Wheat DH Populations
Source: Int J Mol Sci. 2021 Nov 3;22(21):11934. doi: 10.3390/ijms222111934 (PMC8585063; doi:10.3390/ijms222111934)
Supplement: Supplementary file 1 [file ijms-22-11934-s001.zip › BW SpB Supplimental Figures-20211102.pptx]

## Slide 1
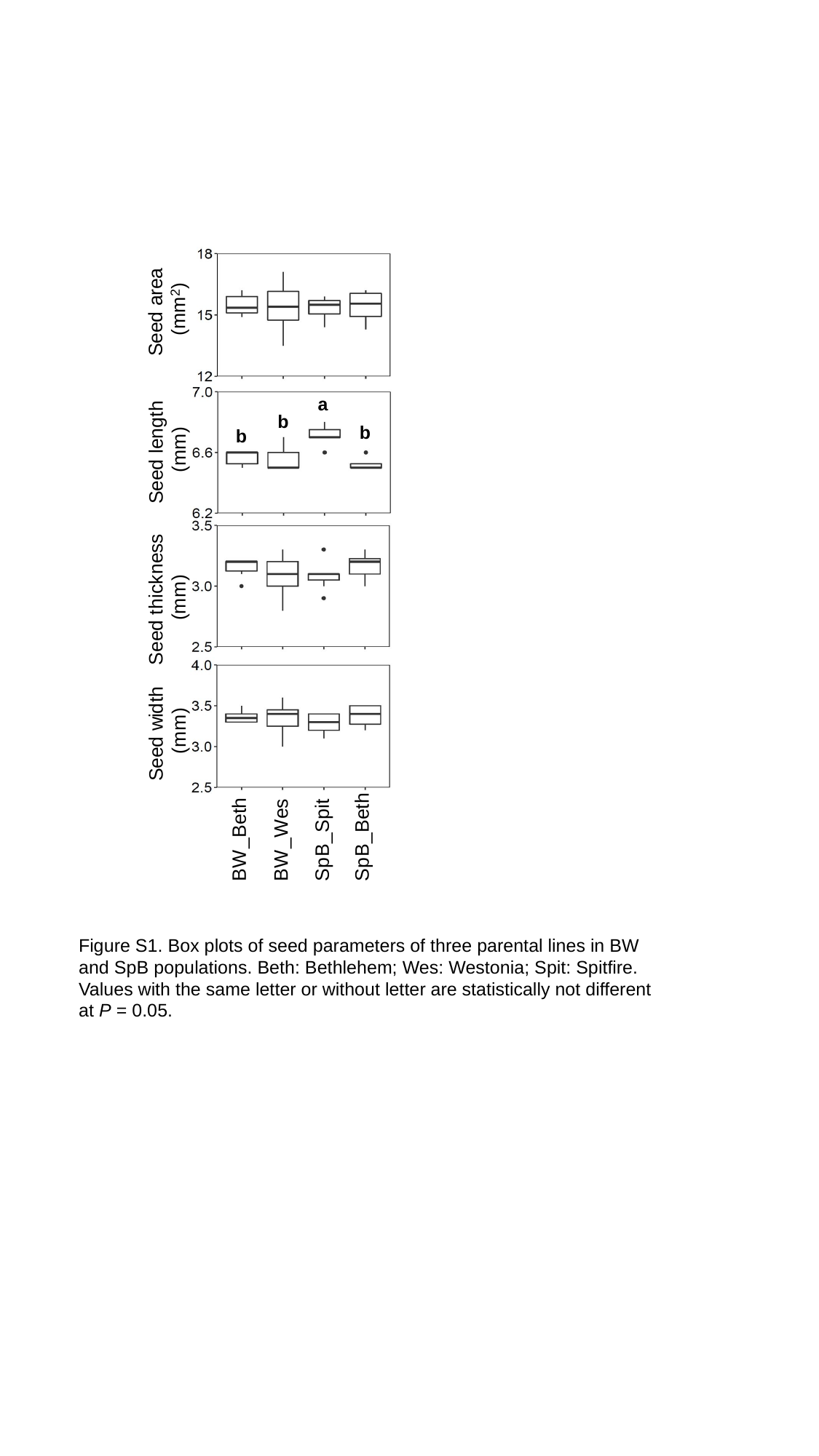

Figure S1. Box plots of seed parameters of three parental lines in BW and SpB populations. Beth: Bethlehem; Wes: Westonia; Spit: Spitfire. Values with the same letter or without letter are statistically not different at P = 0.05.

## Slide 2
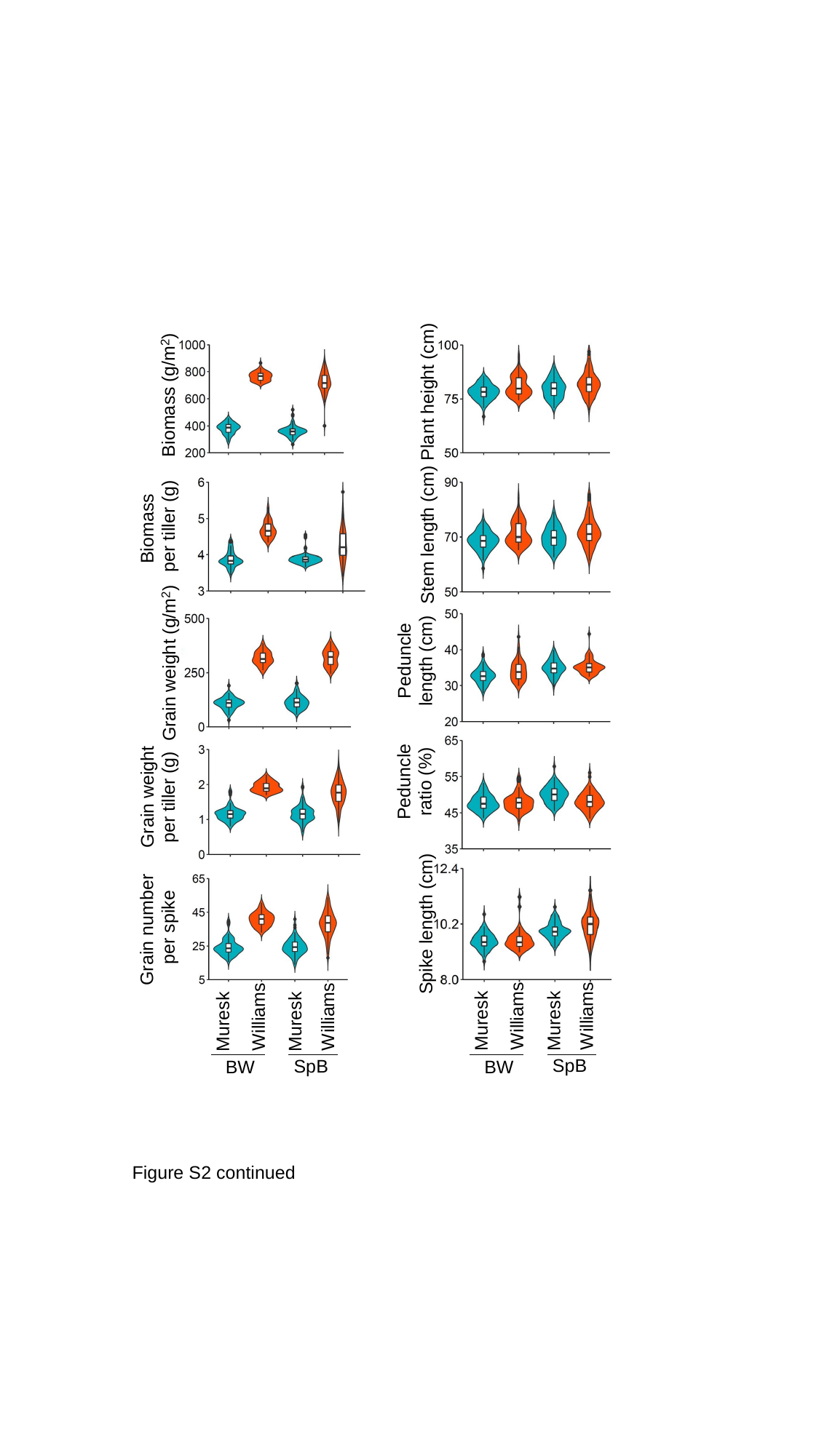

Figure S2 continued

## Slide 3
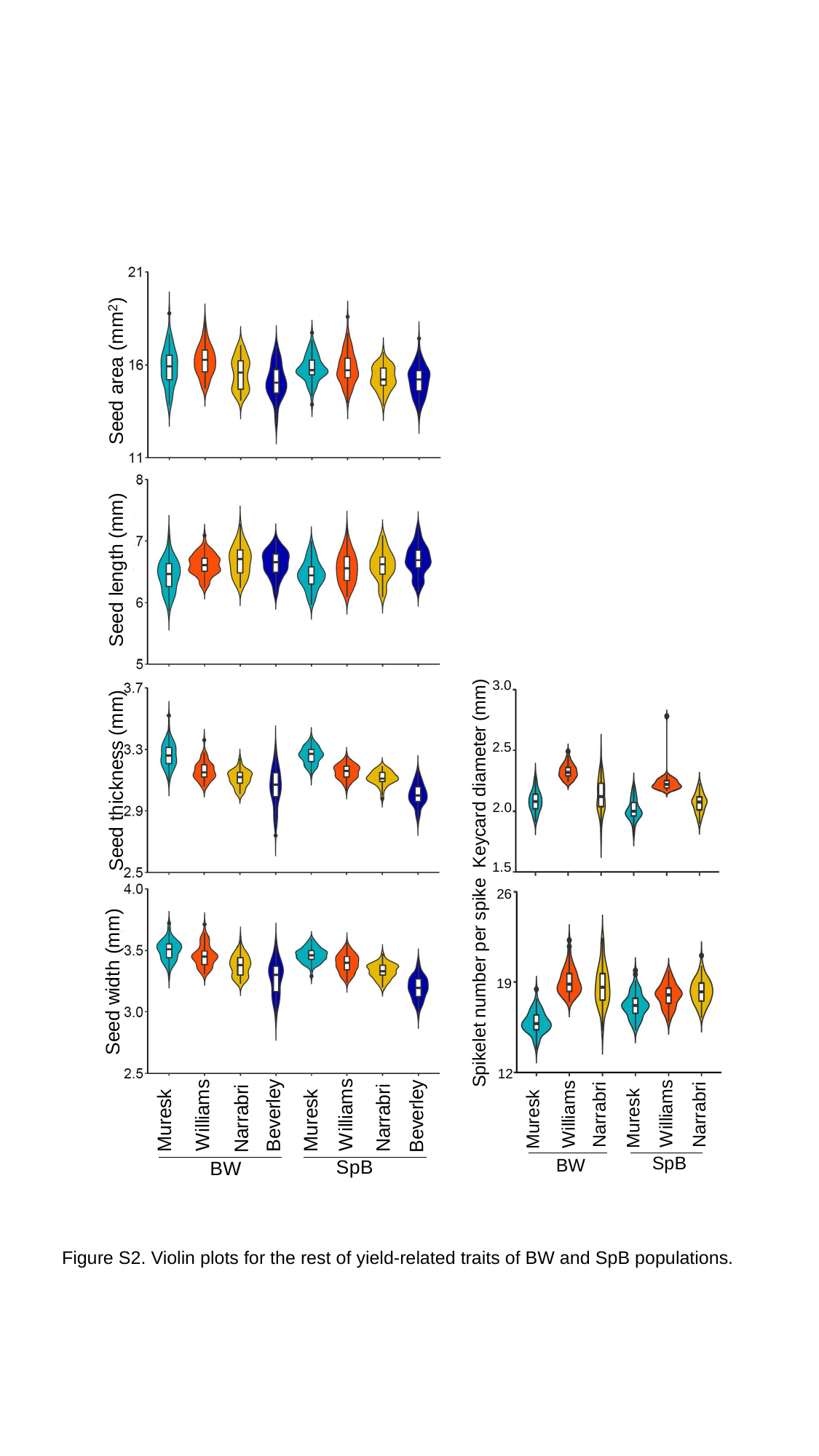

Figure S2. Violin plots for the rest of yield-related traits of BW and SpB populations.

## Slide 4
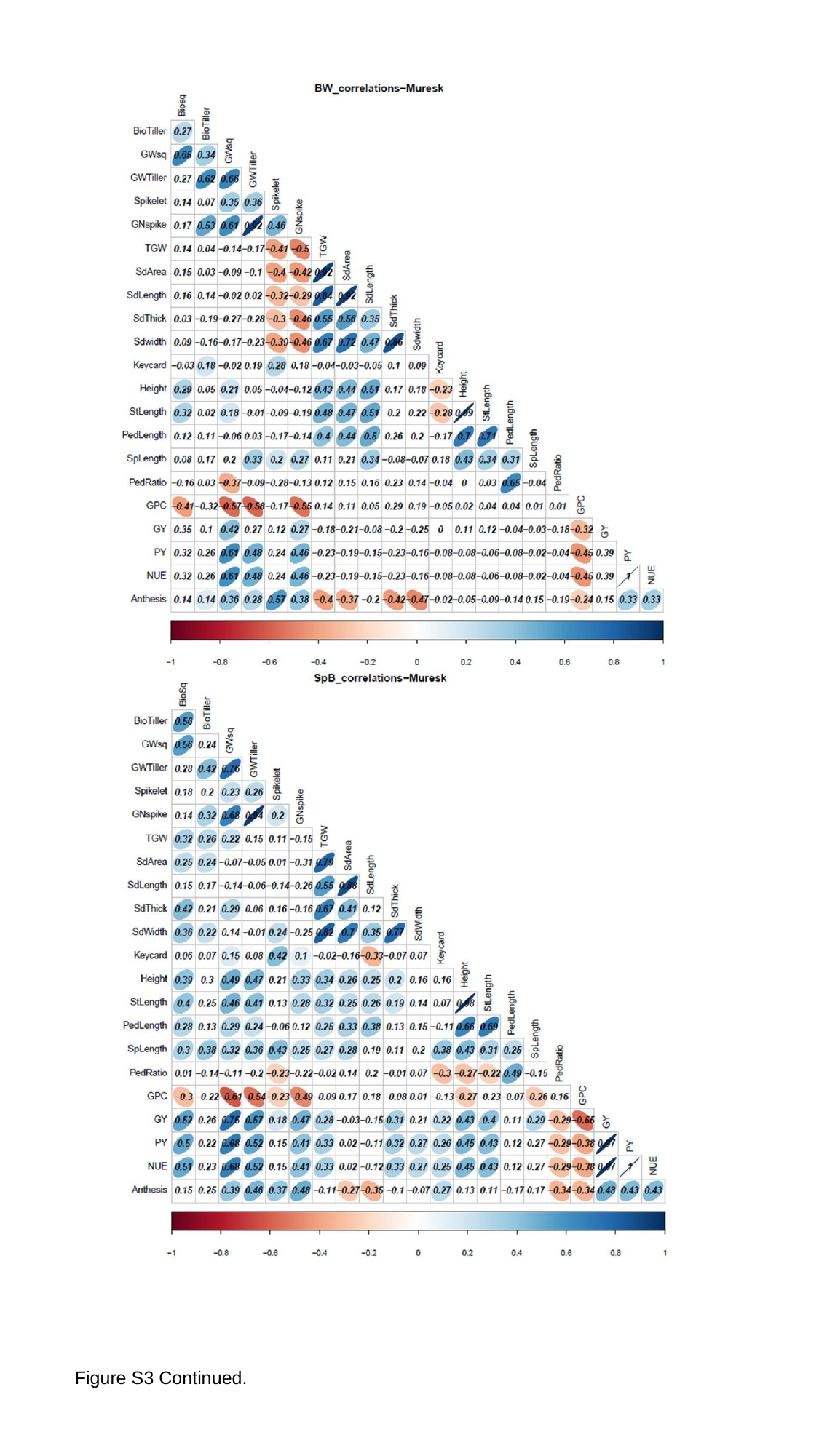

Figure S3 Continued.

## Slide 5
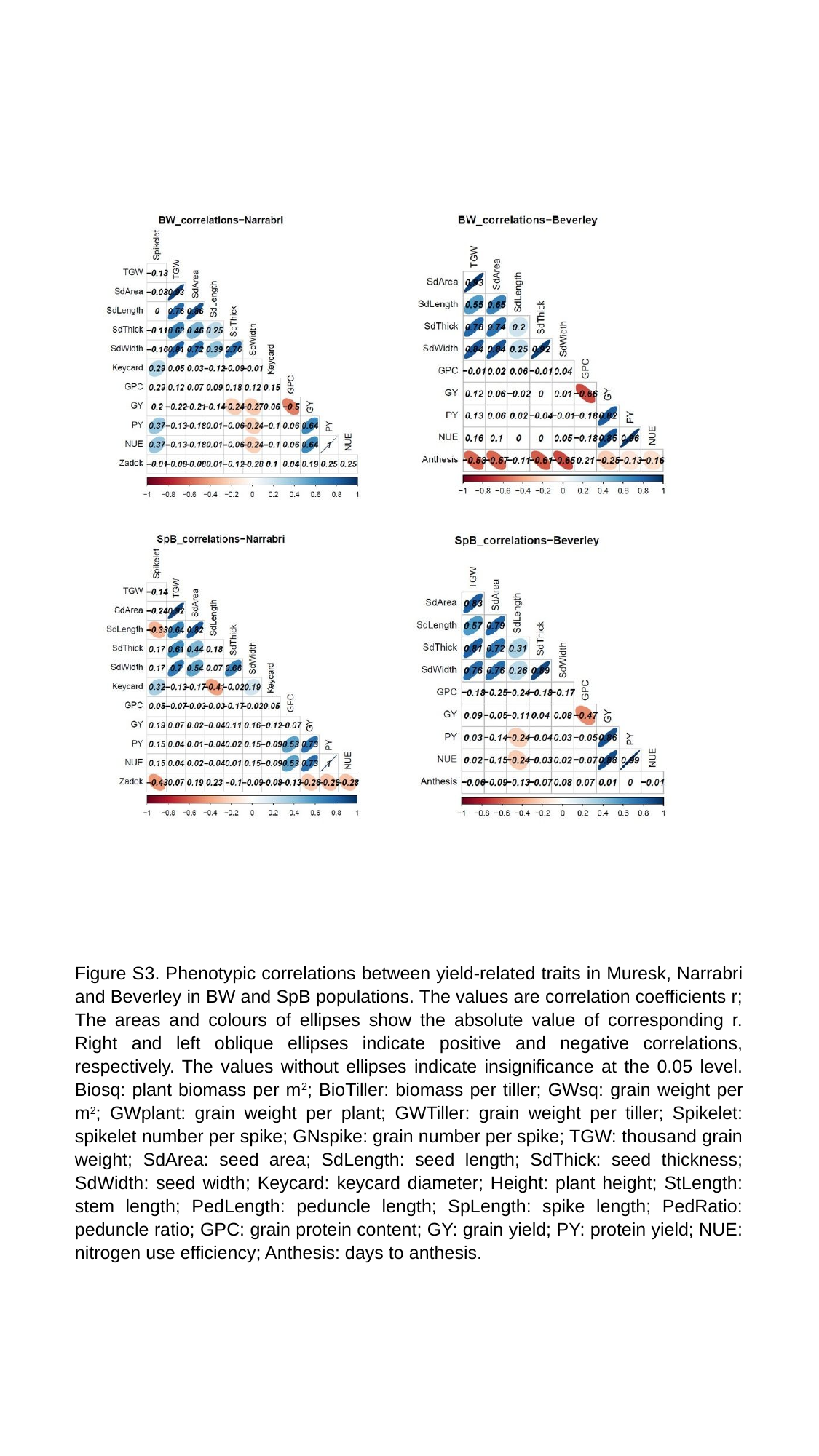

Figure S3. Phenotypic correlations between yield-related traits in Muresk, Narrabri and Beverley in BW and SpB populations. The values are correlation coefficients r; The areas and colours of ellipses show the absolute value of corresponding r. Right and left oblique ellipses indicate positive and negative correlations, respectively. The values without ellipses indicate insignificance at the 0.05 level. Biosq: plant biomass per m2; BioTiller: biomass per tiller; GWsq: grain weight per m2; GWplant: grain weight per plant; GWTiller: grain weight per tiller; Spikelet: spikelet number per spike; GNspike: grain number per spike; TGW: thousand grain weight; SdArea: seed area; SdLength: seed length; SdThick: seed thickness; SdWidth: seed width; Keycard: keycard diameter; Height: plant height; StLength: stem length; PedLength: peduncle length; SpLength: spike length; PedRatio: peduncle ratio; GPC: grain protein content; GY: grain yield; PY: protein yield; NUE: nitrogen use efficiency; Anthesis: days to anthesis.

## Slide 6
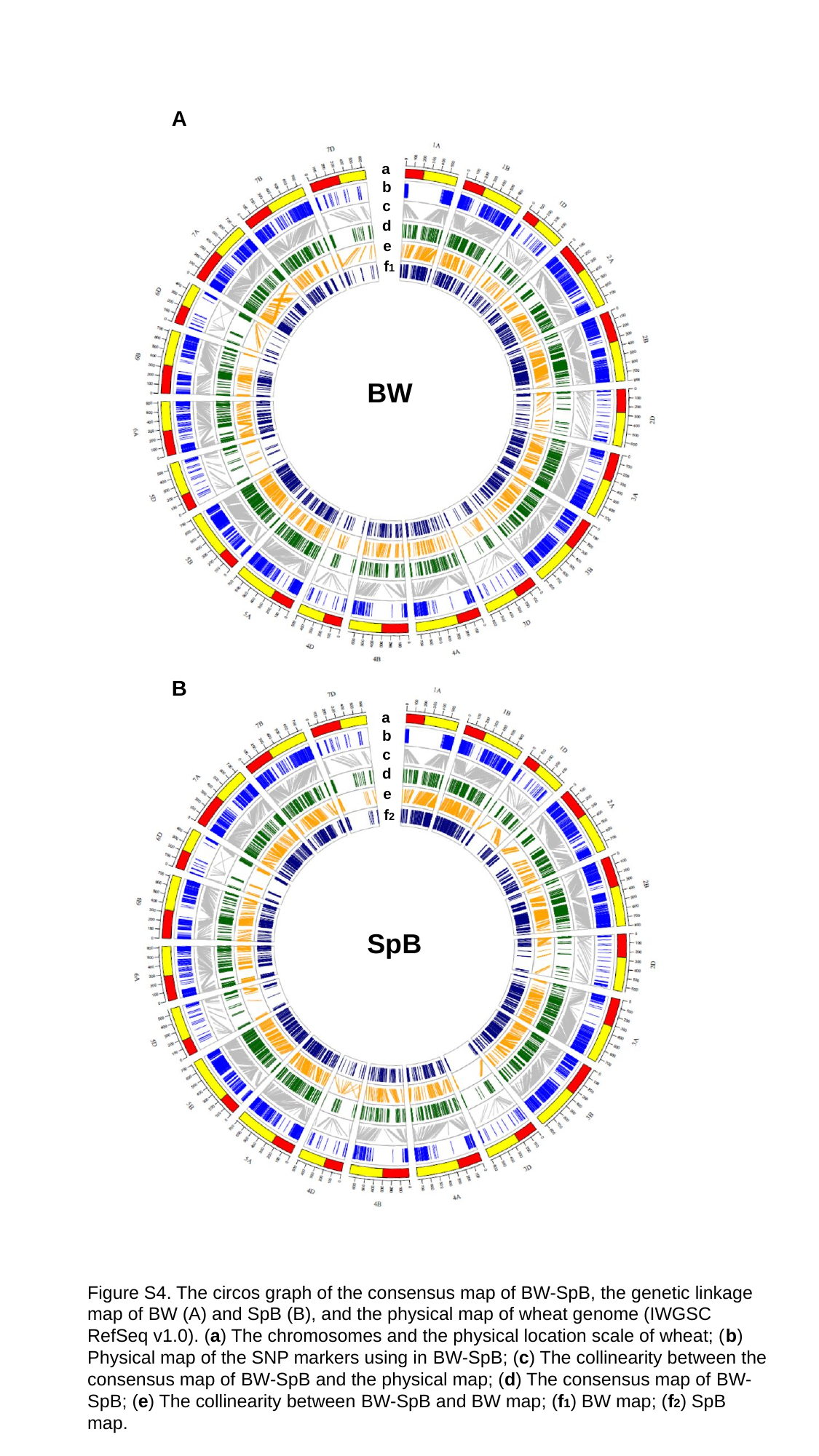

Figure S4. The circos graph of the consensus map of BW-SpB, the genetic linkage map of BW (A) and SpB (B), and the physical map of wheat genome (IWGSC RefSeq v1.0). (a) The chromosomes and the physical location scale of wheat; (b) Physical map of the SNP markers using in BW-SpB; (c) The collinearity between the consensus map of BW-SpB and the physical map; (d) The consensus map of BW-SpB; (e) The collinearity between BW-SpB and BW map; (f1) BW map; (f2) SpB map.

## Slide 7
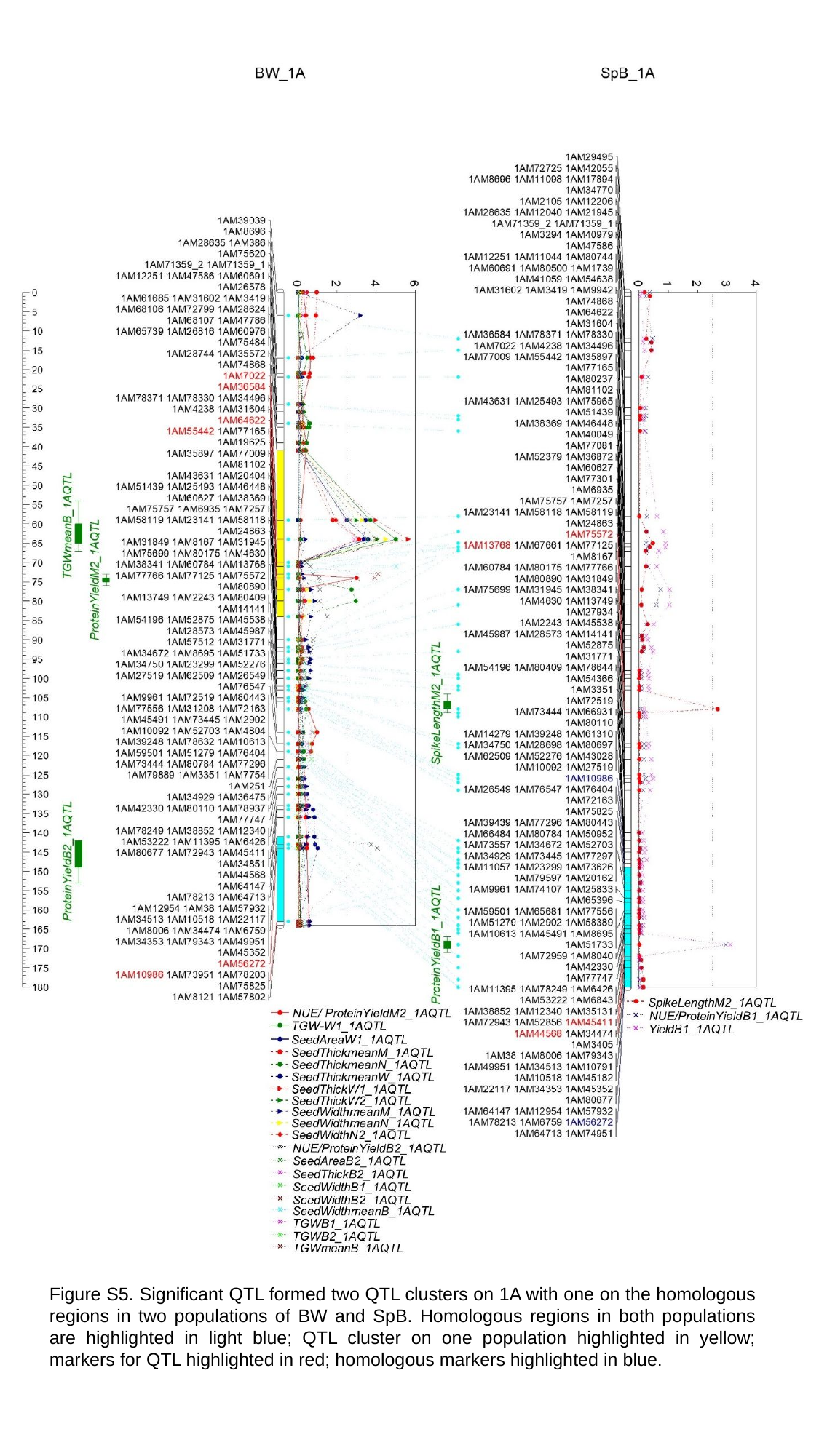

Figure S5. Significant QTL formed two QTL clusters on 1A with one on the homologous regions in two populations of BW and SpB. Homologous regions in both populations are highlighted in light blue; QTL cluster on one population highlighted in yellow; markers for QTL highlighted in red; homologous markers highlighted in blue.

## Slide 8
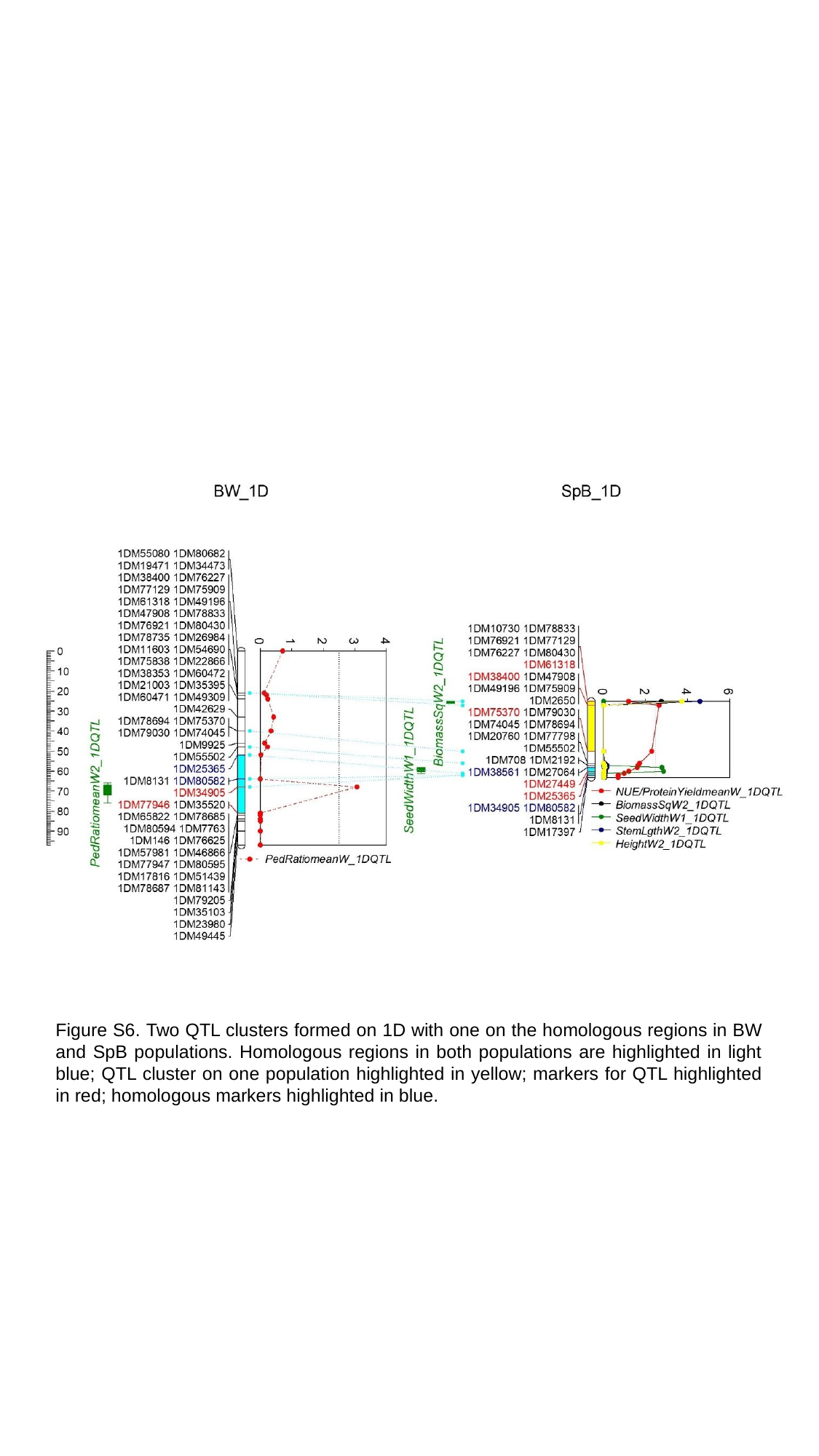

Figure S6. Two QTL clusters formed on 1D with one on the homologous regions in BW and SpB populations. Homologous regions in both populations are highlighted in light blue; QTL cluster on one population highlighted in yellow; markers for QTL highlighted in red; homologous markers highlighted in blue.

## Slide 9
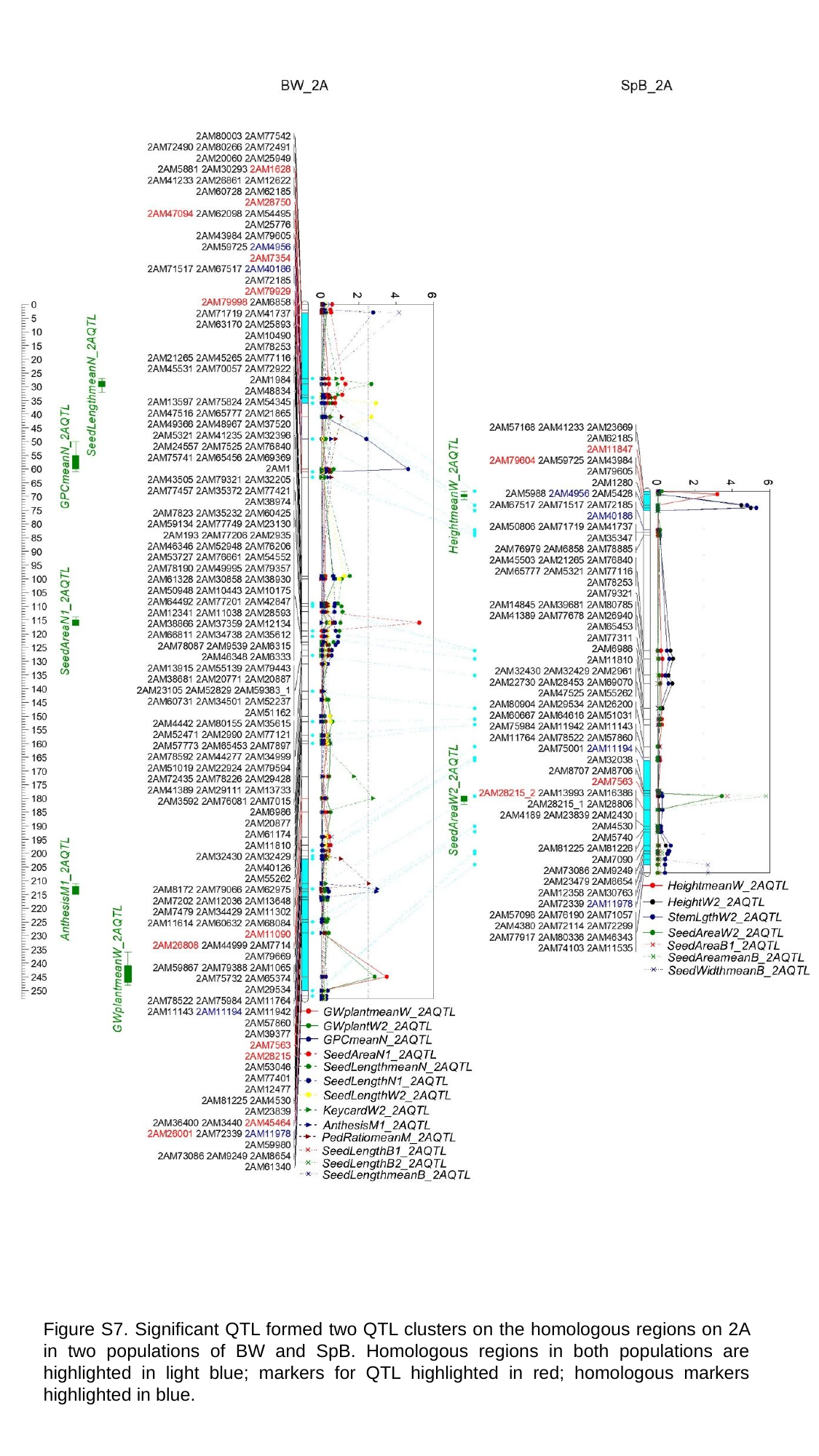

Figure S7. Significant QTL formed two QTL clusters on the homologous regions on 2A in two populations of BW and SpB. Homologous regions in both populations are highlighted in light blue; markers for QTL highlighted in red; homologous markers highlighted in blue.

## Slide 10
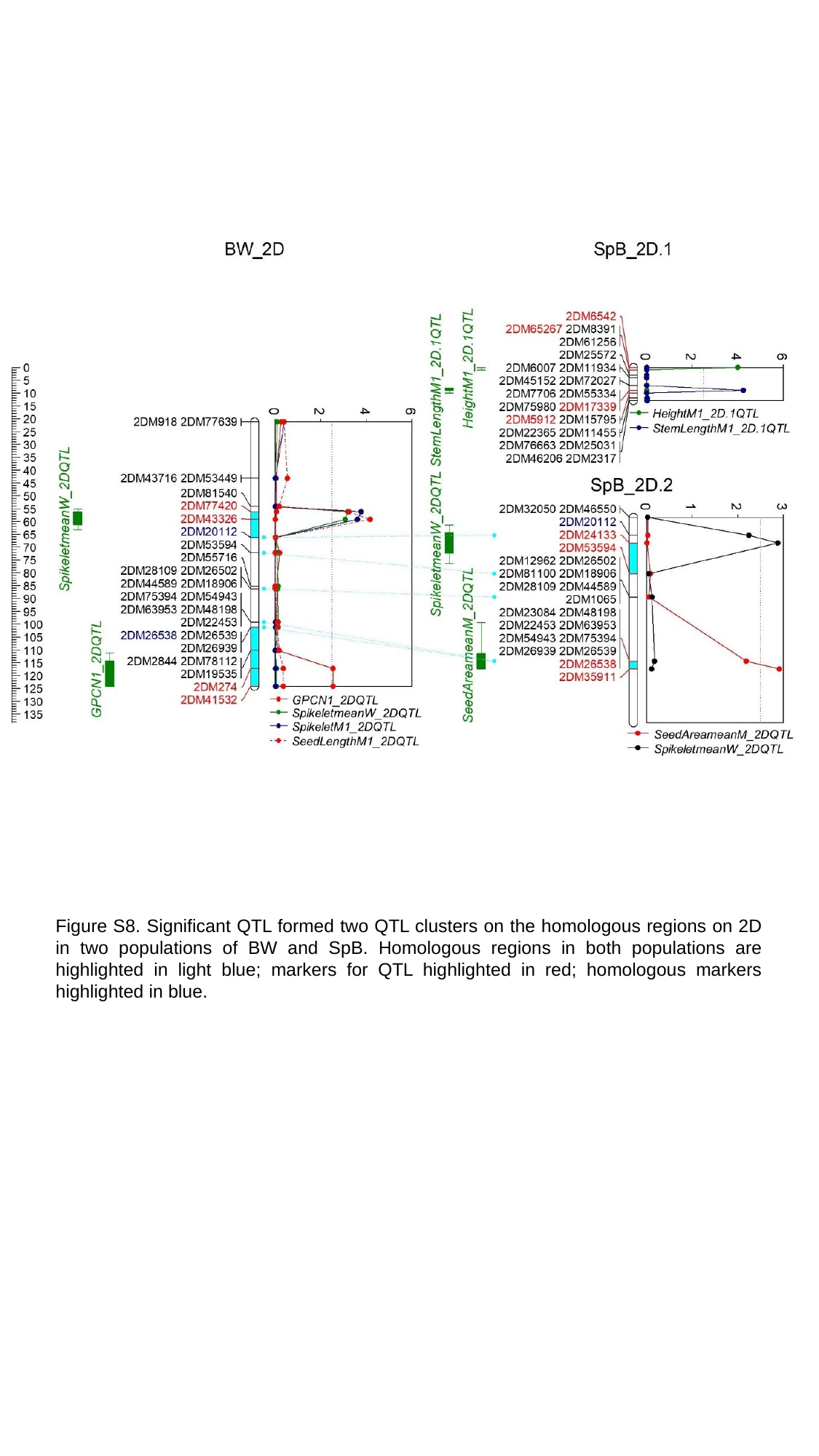

Figure S8. Significant QTL formed two QTL clusters on the homologous regions on 2D in two populations of BW and SpB. Homologous regions in both populations are highlighted in light blue; markers for QTL highlighted in red; homologous markers highlighted in blue.

## Slide 11
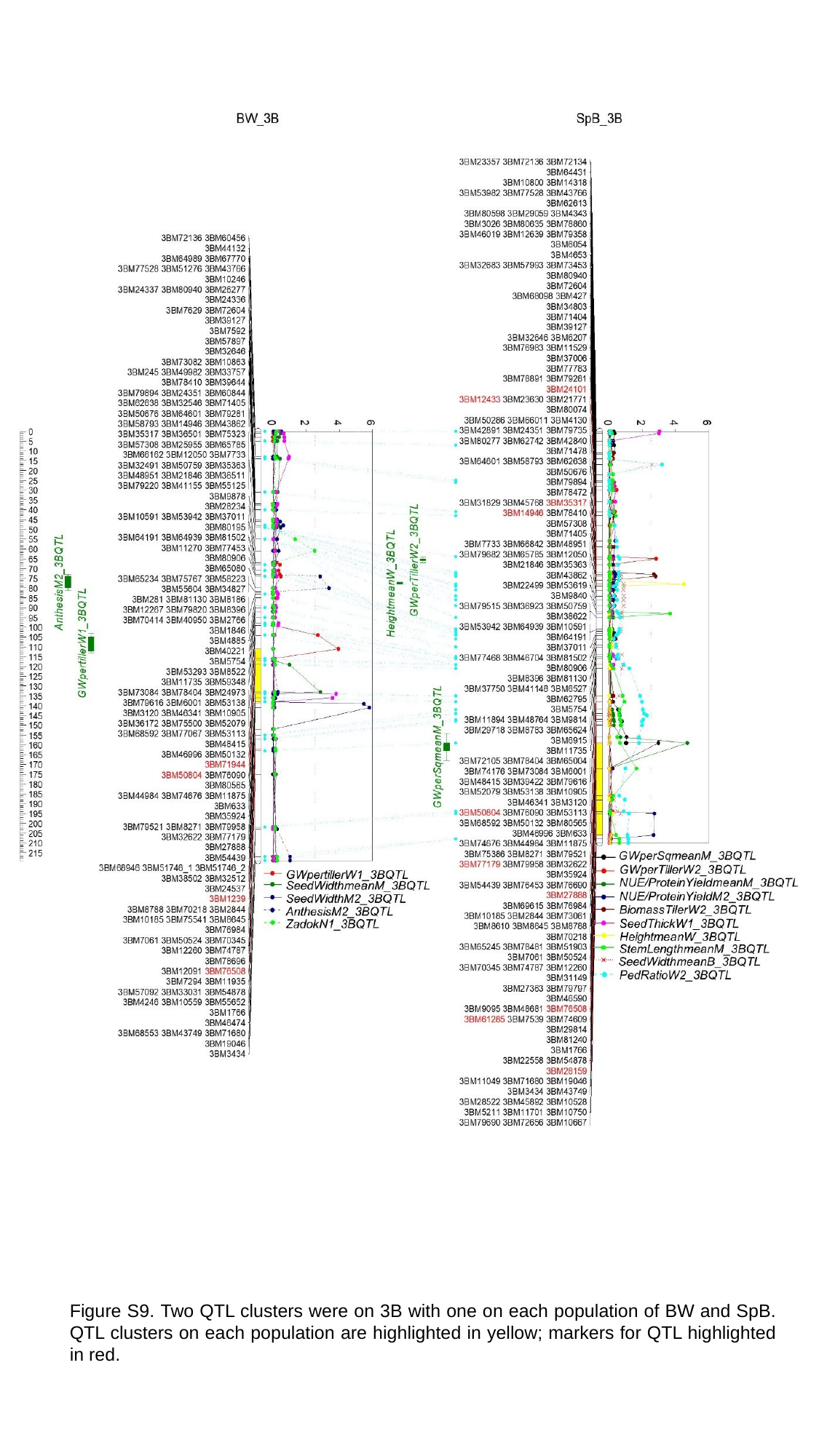

Figure S9. Two QTL clusters were on 3B with one on each population of BW and SpB. QTL clusters on each population are highlighted in yellow; markers for QTL highlighted in red.

## Slide 12
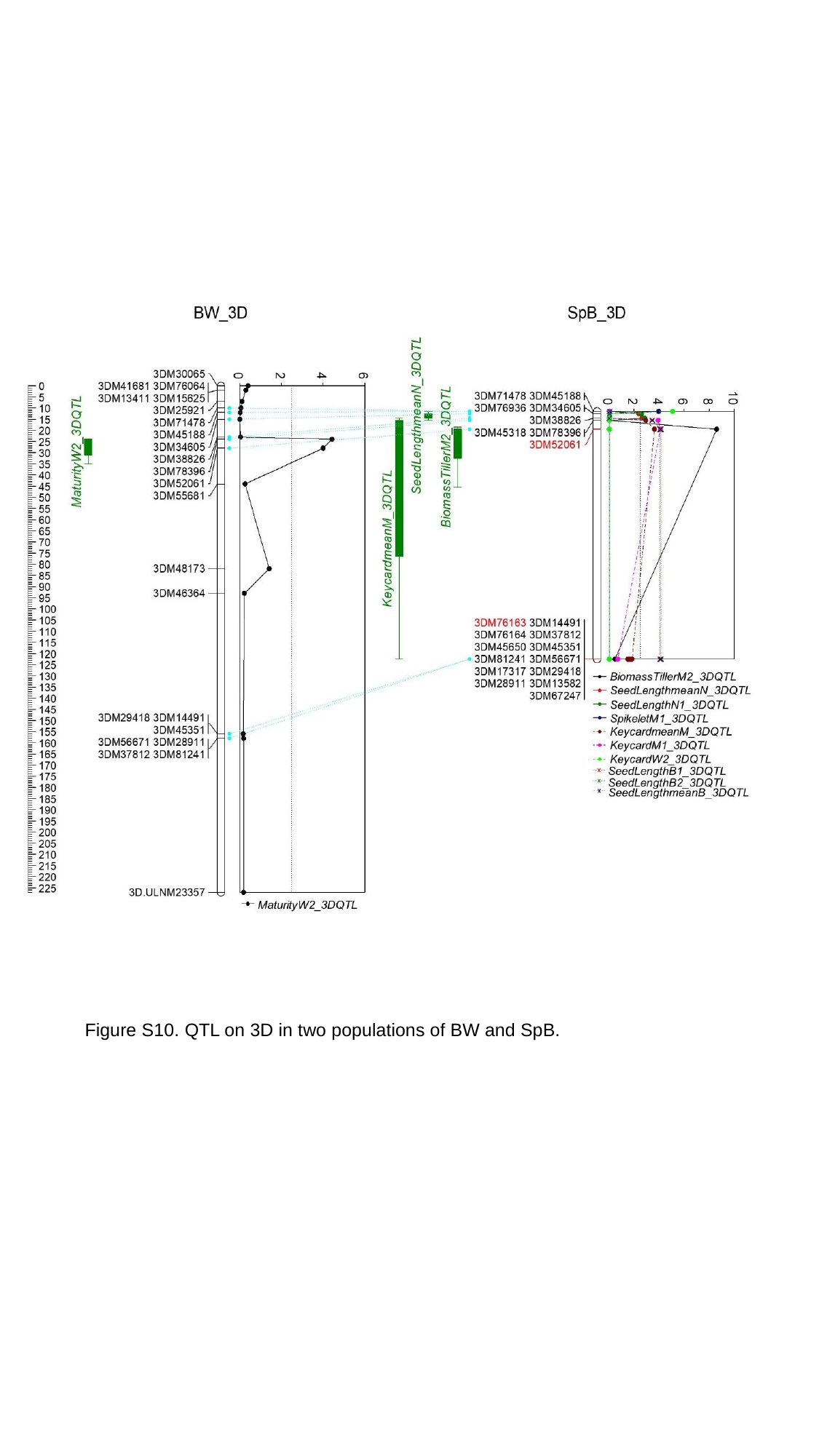

Figure S10. QTL on 3D in two populations of BW and SpB.

## Slide 13
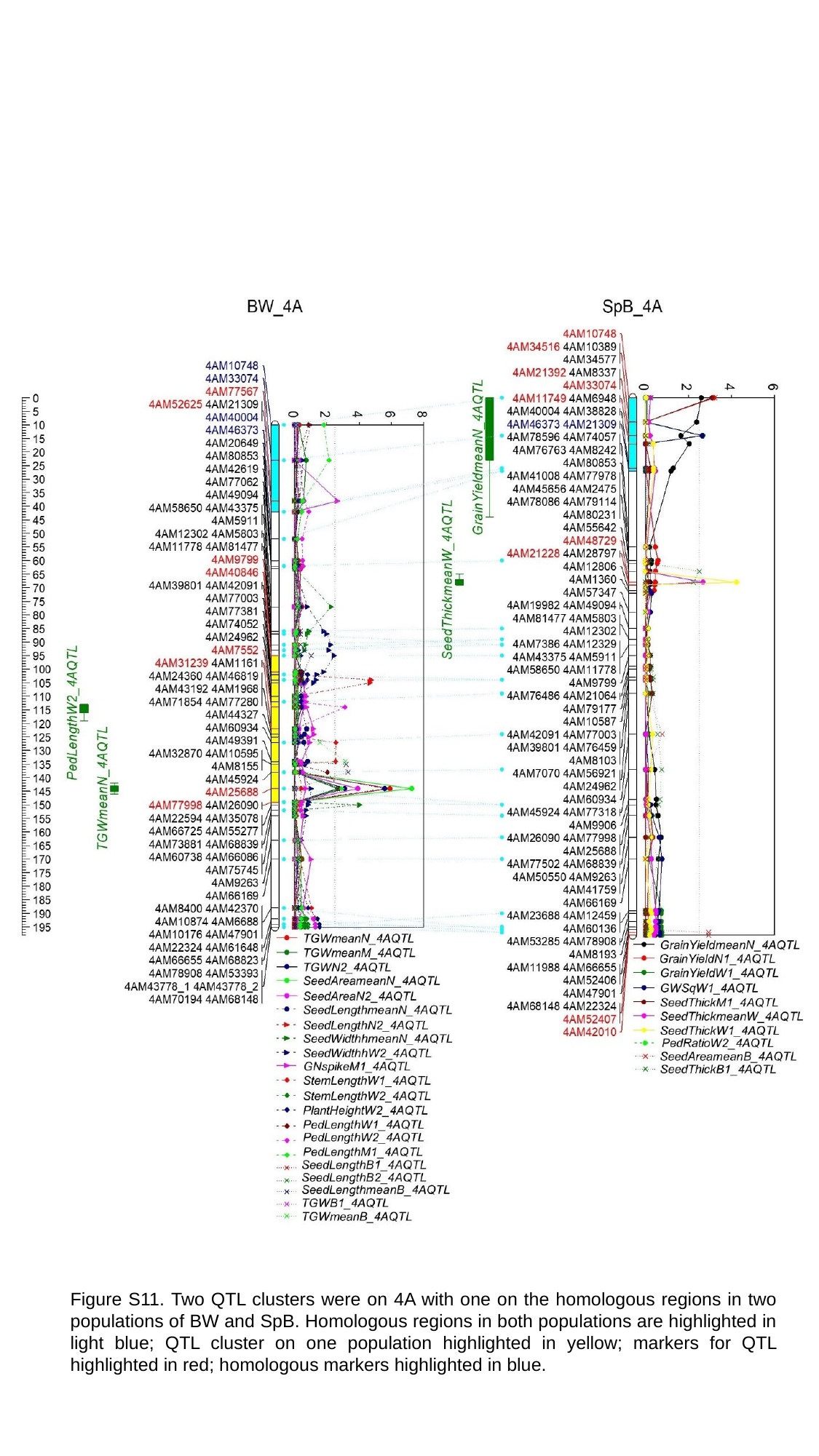

Figure S11. Two QTL clusters were on 4A with one on the homologous regions in two populations of BW and SpB. Homologous regions in both populations are highlighted in light blue; QTL cluster on one population highlighted in yellow; markers for QTL highlighted in red; homologous markers highlighted in blue.

## Slide 14
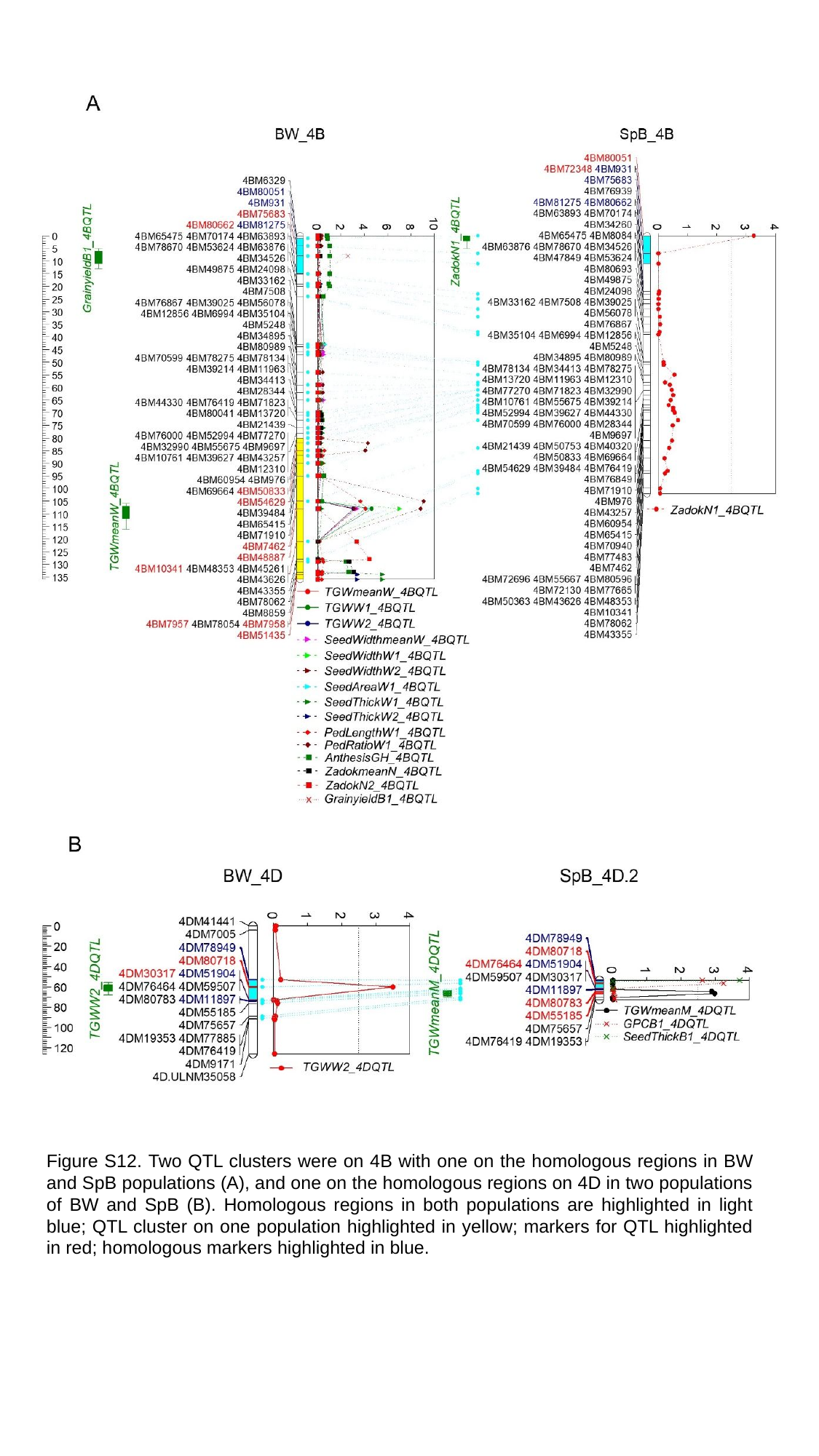

Figure S12. Two QTL clusters were on 4B with one on the homologous regions in BW and SpB populations (A), and one on the homologous regions on 4D in two populations of BW and SpB (B). Homologous regions in both populations are highlighted in light blue; QTL cluster on one population highlighted in yellow; markers for QTL highlighted in red; homologous markers highlighted in blue.

## Slide 15
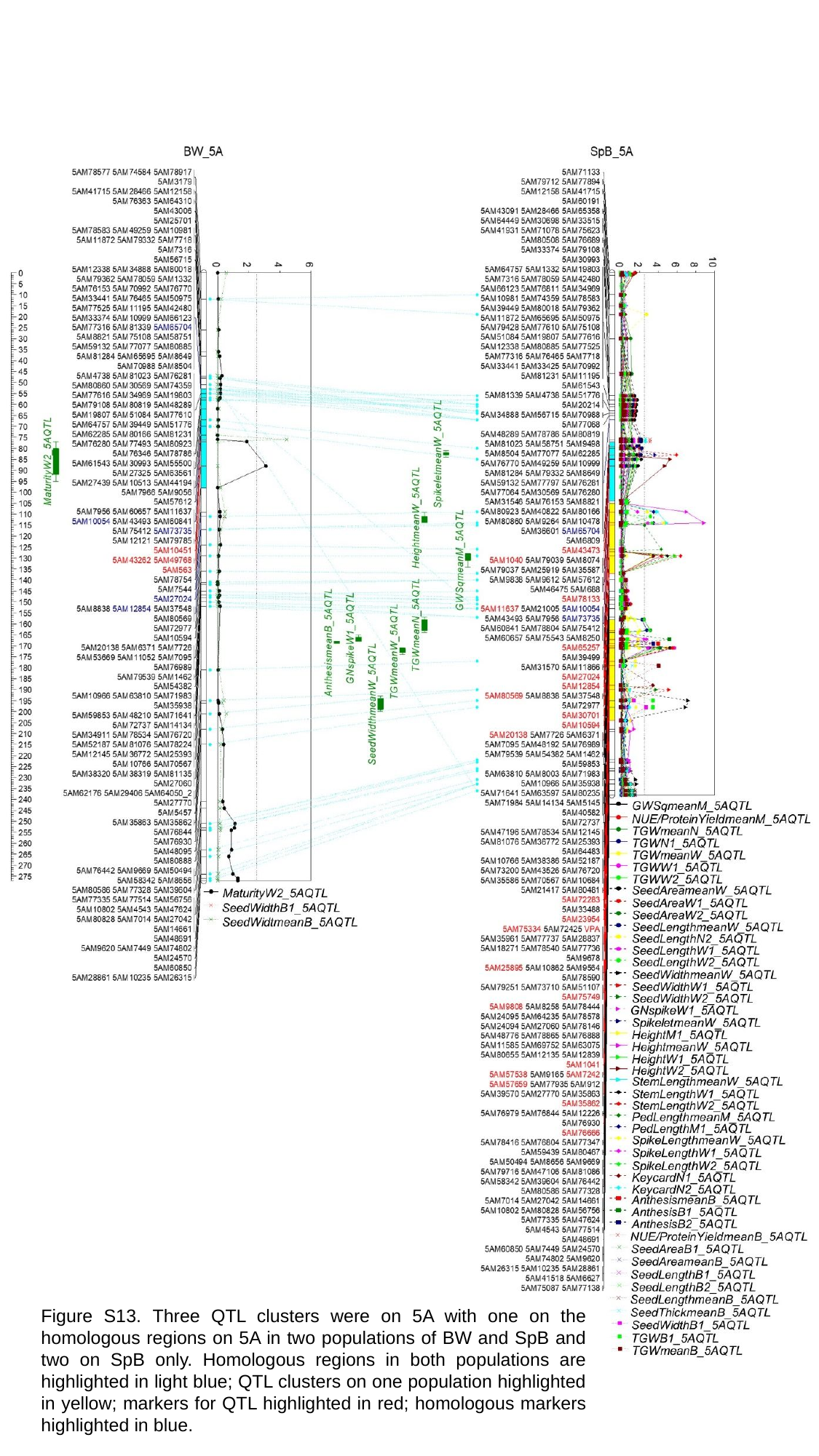

Figure S13. Three QTL clusters were on 5A with one on the homologous regions on 5A in two populations of BW and SpB and two on SpB only. Homologous regions in both populations are highlighted in light blue; QTL clusters on one population highlighted in yellow; markers for QTL highlighted in red; homologous markers highlighted in blue.

## Slide 16
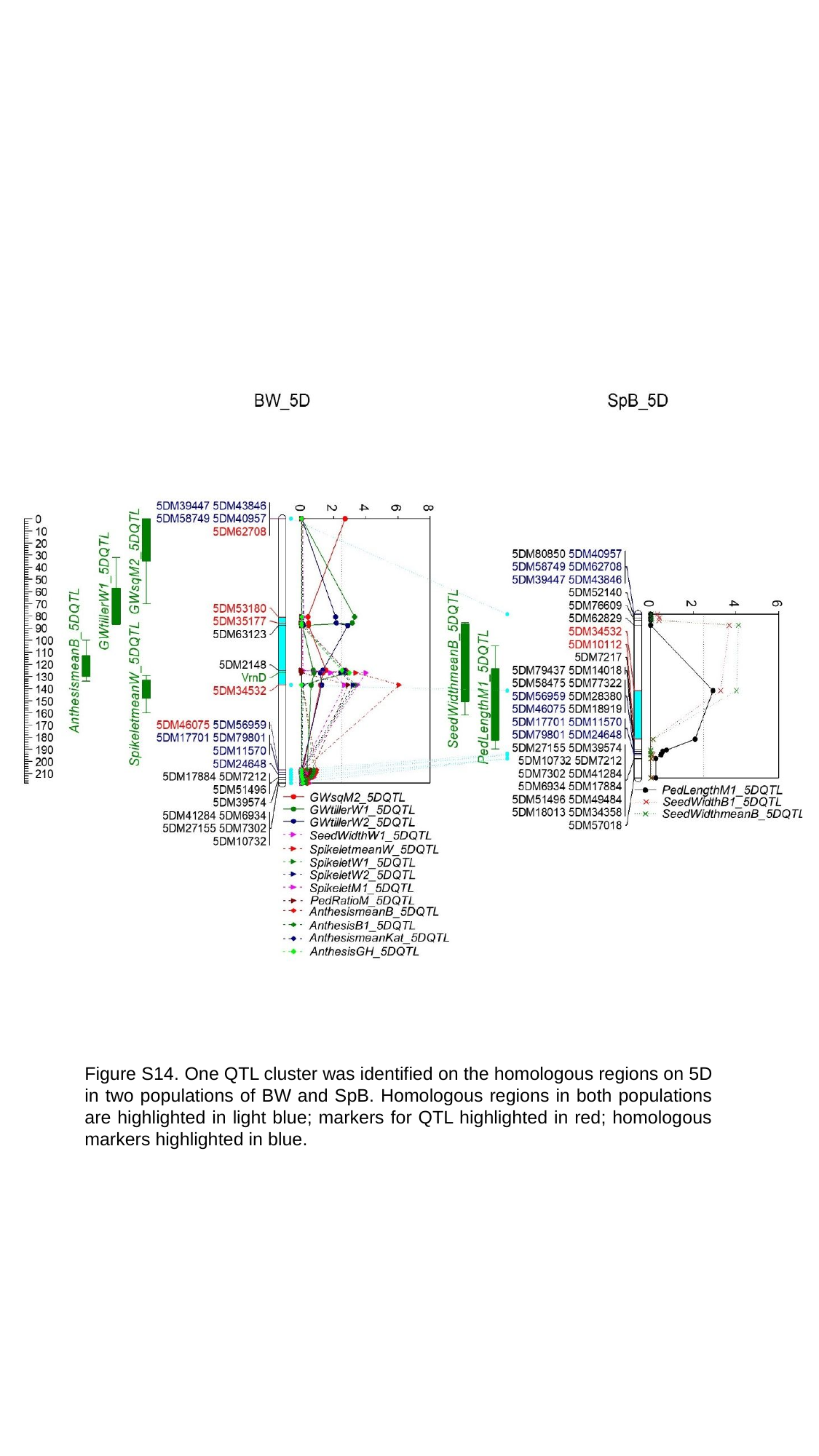

Figure S14. One QTL cluster was identified on the homologous regions on 5D in two populations of BW and SpB. Homologous regions in both populations are highlighted in light blue; markers for QTL highlighted in red; homologous markers highlighted in blue.

## Slide 17
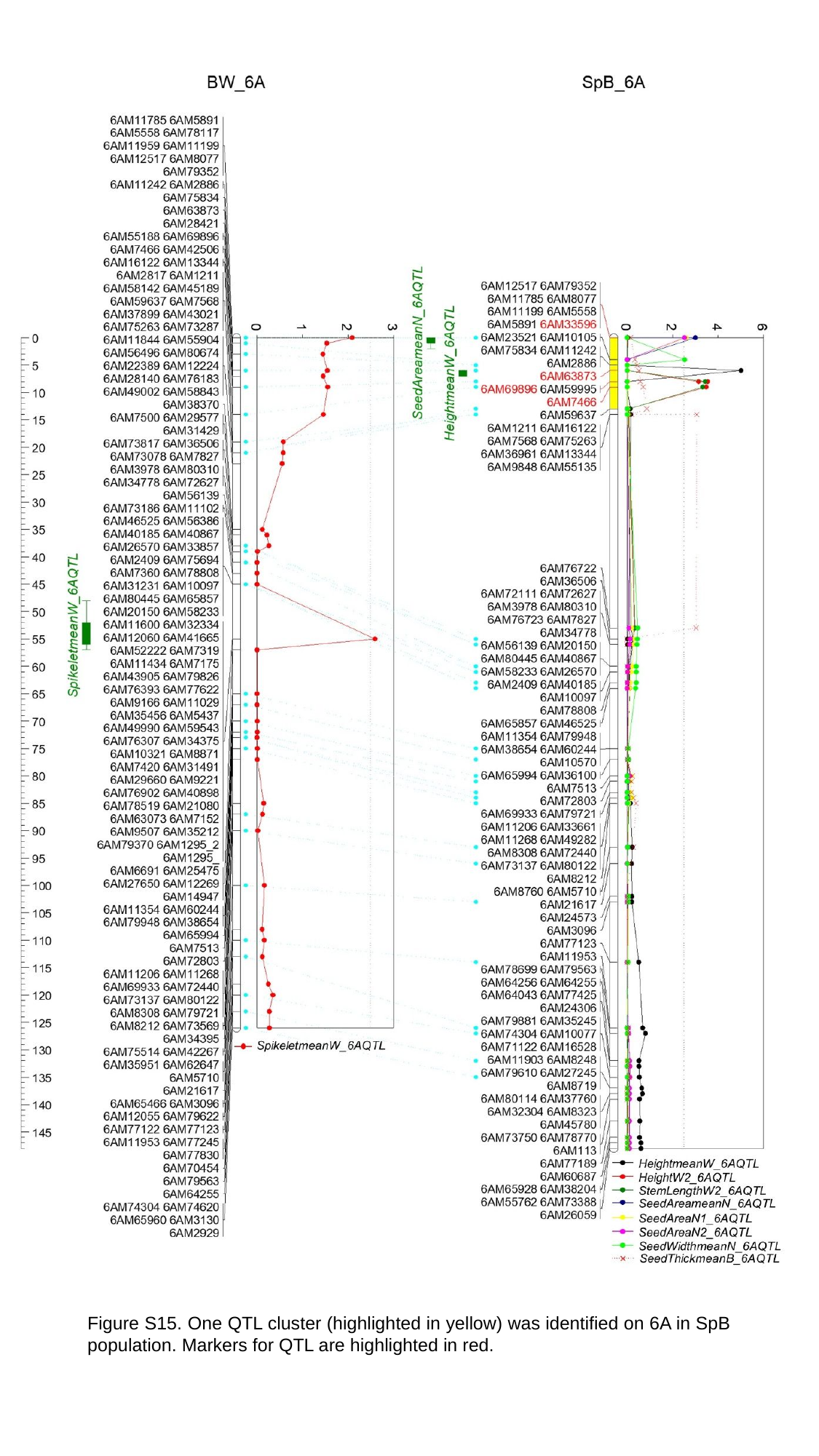

Figure S15. One QTL cluster (highlighted in yellow) was identified on 6A in SpB population. Markers for QTL are highlighted in red.

## Slide 18
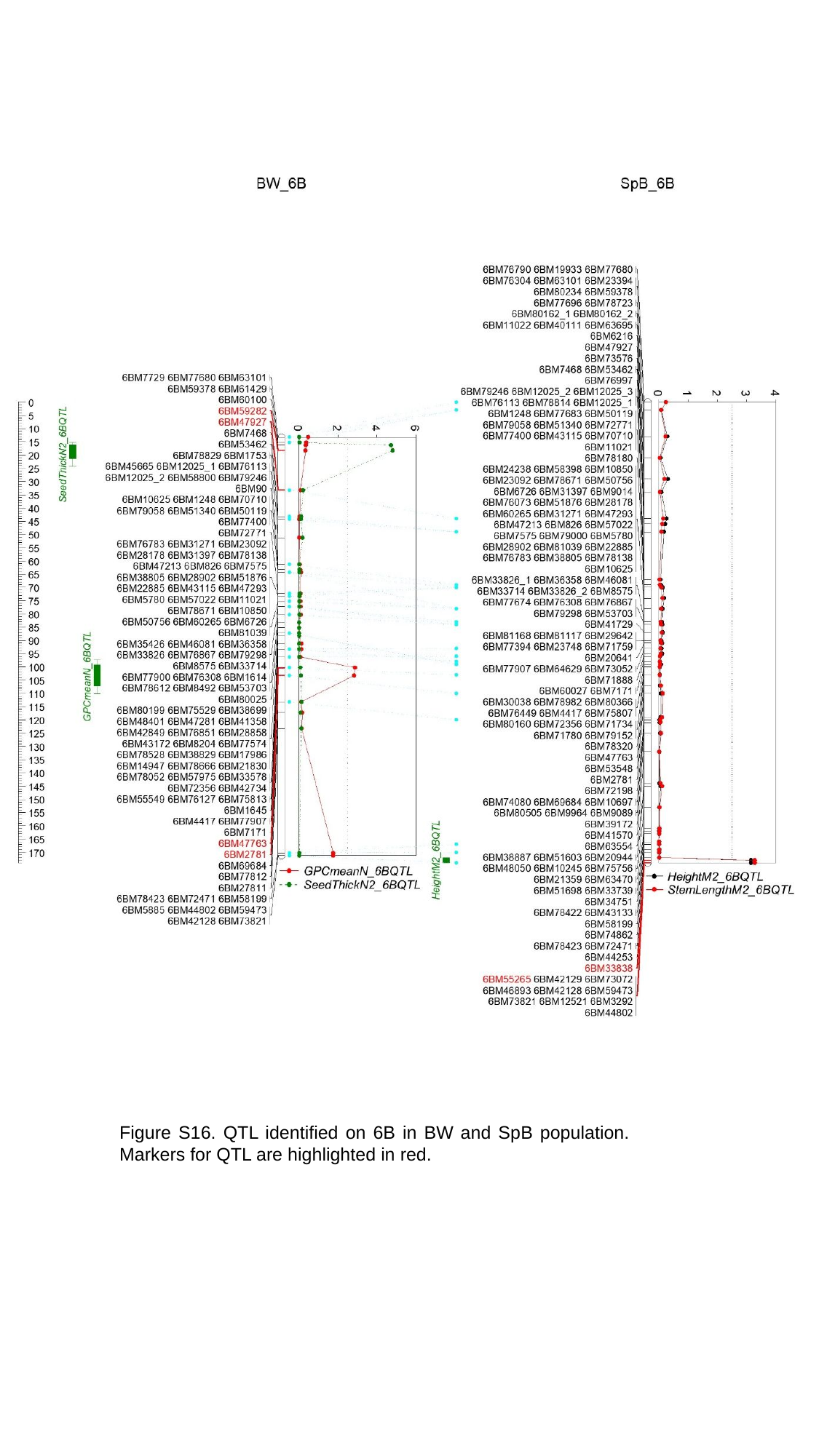

Figure S16. QTL identified on 6B in BW and SpB population. Markers for QTL are highlighted in red.

## Slide 19
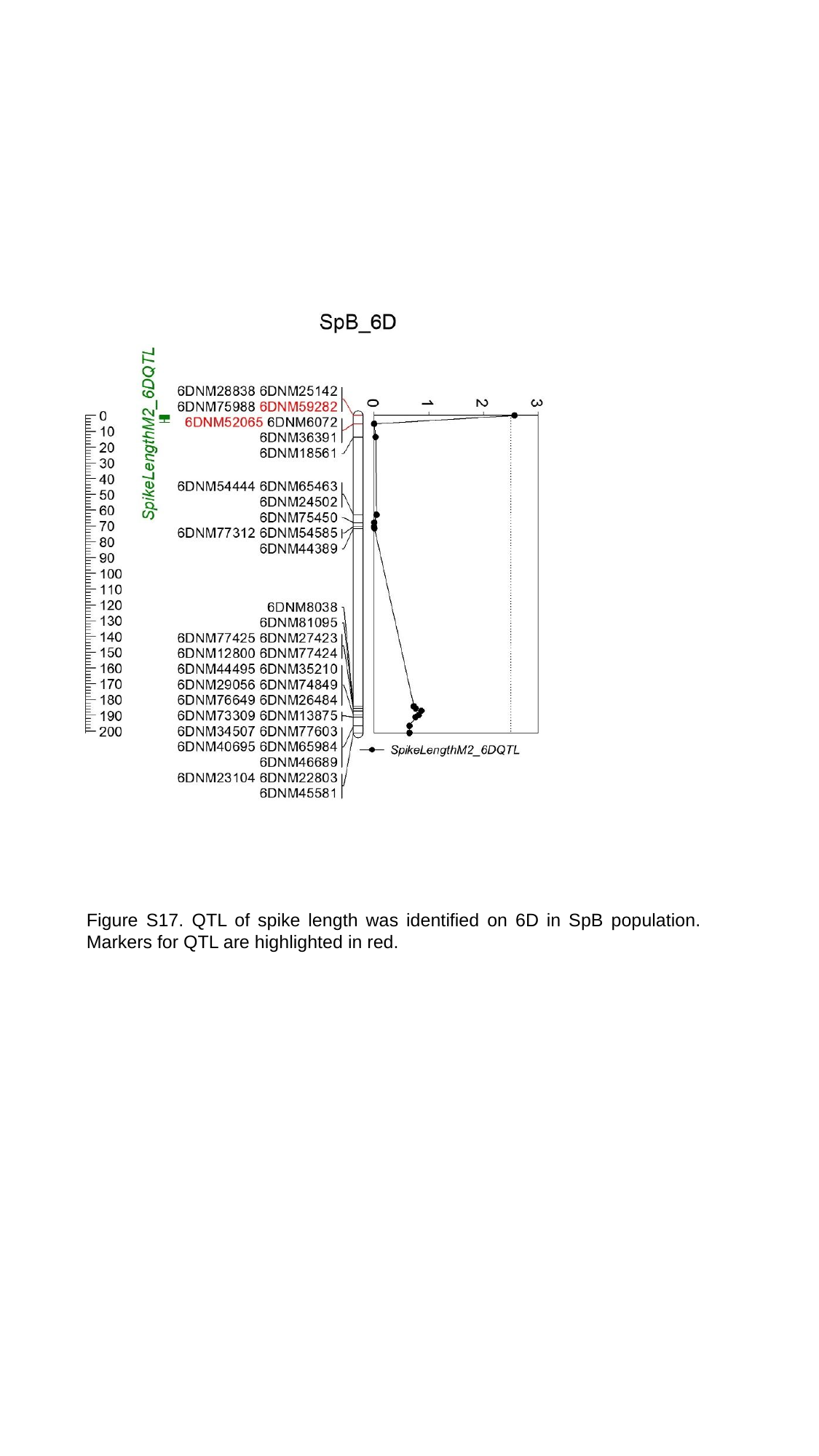

Figure S17. QTL of spike length was identified on 6D in SpB population. Markers for QTL are highlighted in red.

## Slide 20
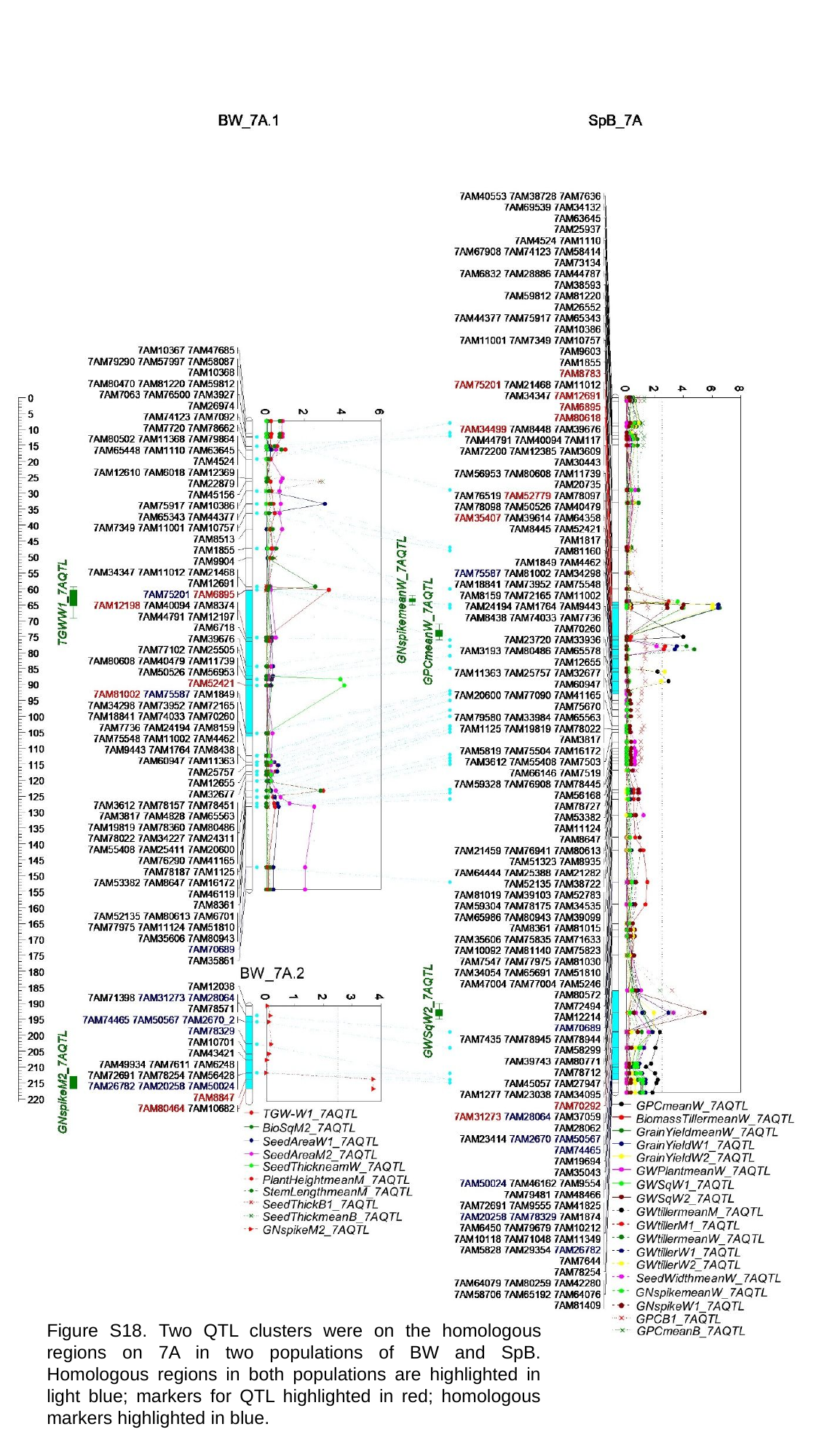

Figure S18. Two QTL clusters were on the homologous regions on 7A in two populations of BW and SpB. Homologous regions in both populations are highlighted in light blue; markers for QTL highlighted in red; homologous markers highlighted in blue.

## Slide 21
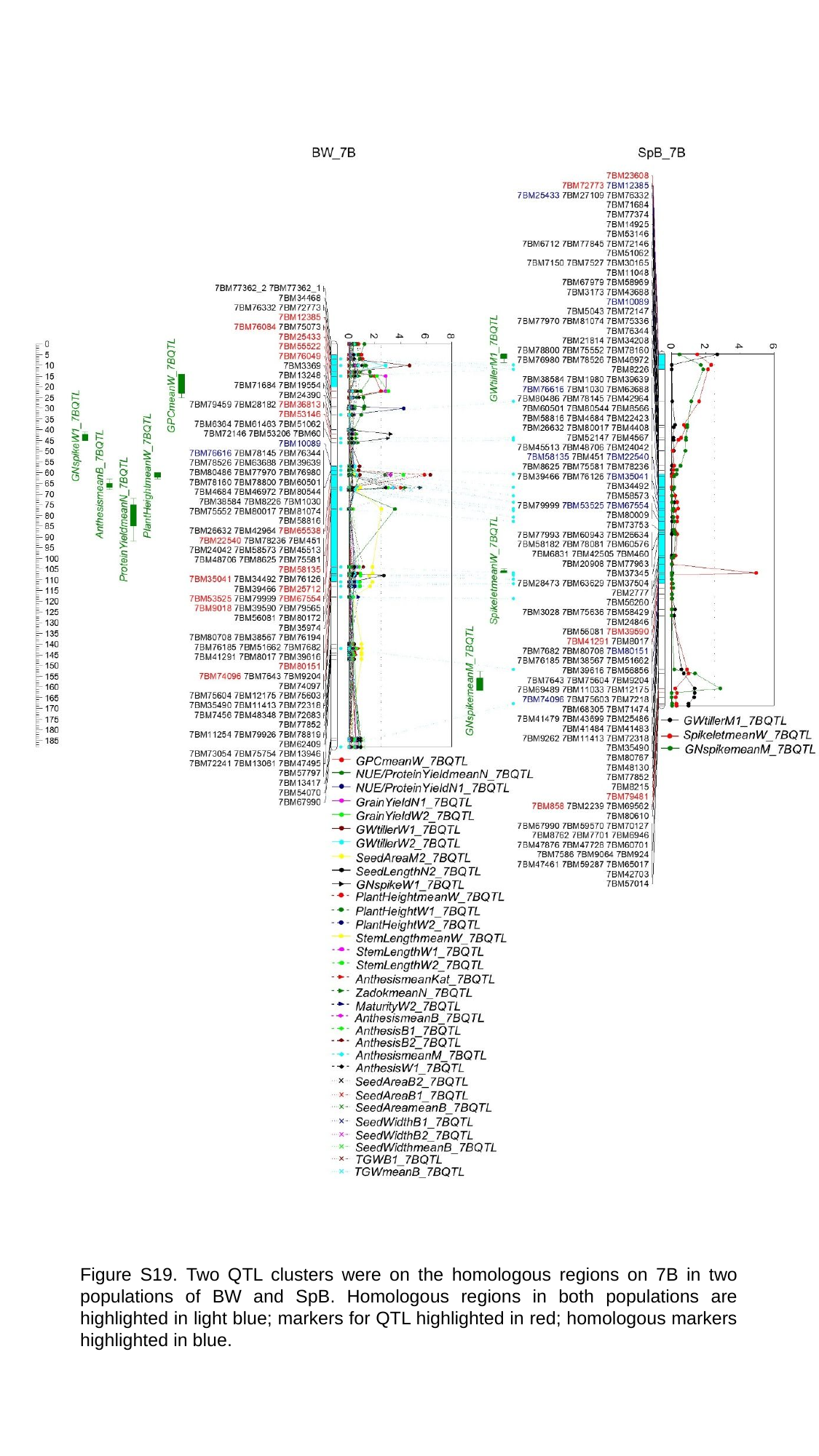

Figure S19. Two QTL clusters were on the homologous regions on 7B in two populations of BW and SpB. Homologous regions in both populations are highlighted in light blue; markers for QTL highlighted in red; homologous markers highlighted in blue.

## Slide 22
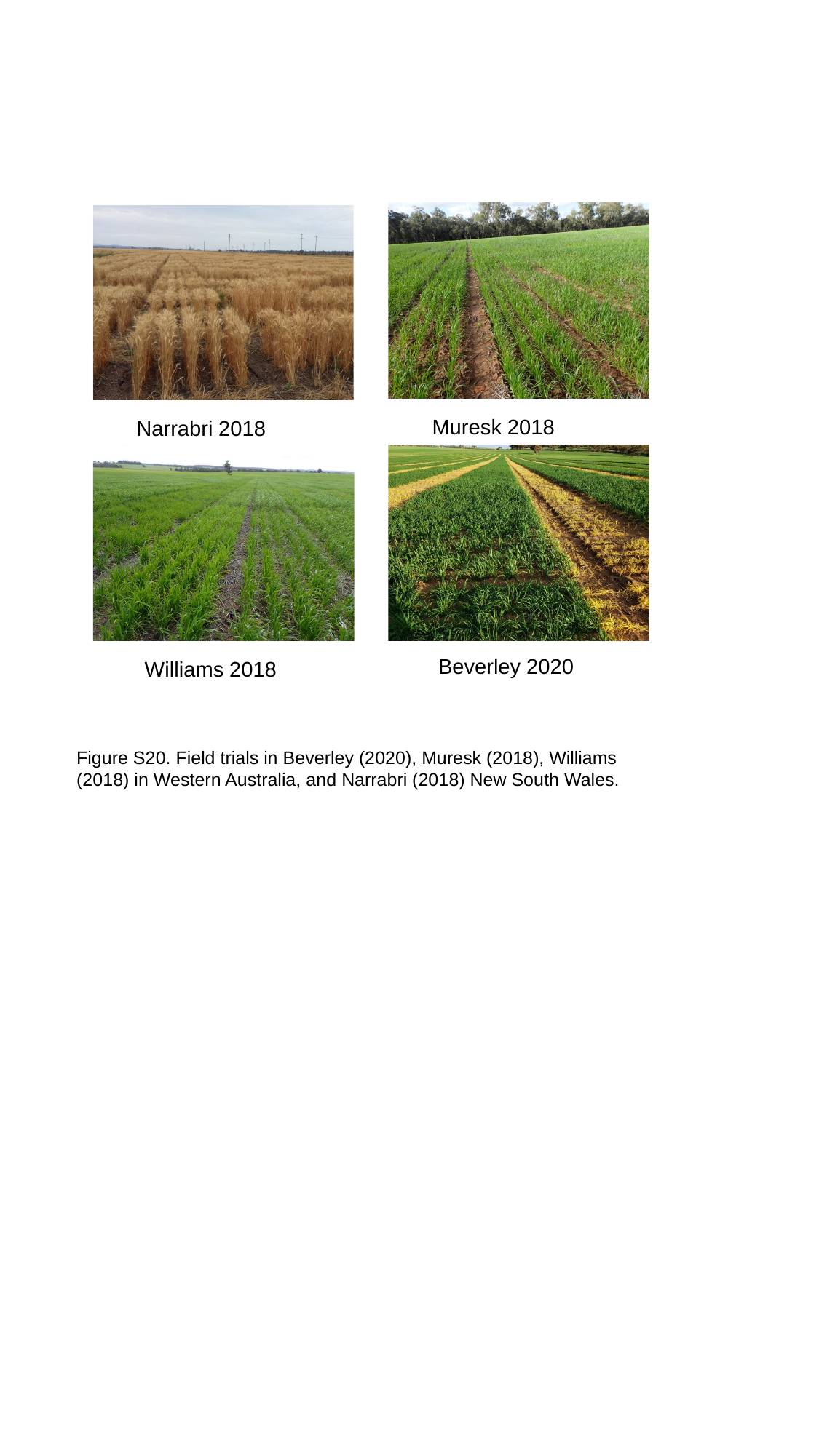

Figure S20. Field trials in Beverley (2020), Muresk (2018), Williams (2018) in Western Australia, and Narrabri (2018) New South Wales.
